# Supplementary material for: CHAMP delivers accurate taxonomic profiles of the prokaryotes, eukaryotes, and bacteriophages in the human microbiome
Source: Front Microbiol. 2024 Oct 17;15:1425489. doi: 10.3389/fmicb.2024.1425489 (PMC11524946; doi:10.3389/fmicb.2024.1425489)
Supplement: Supplementary file 1 [file Data_Sheet_1.docx]

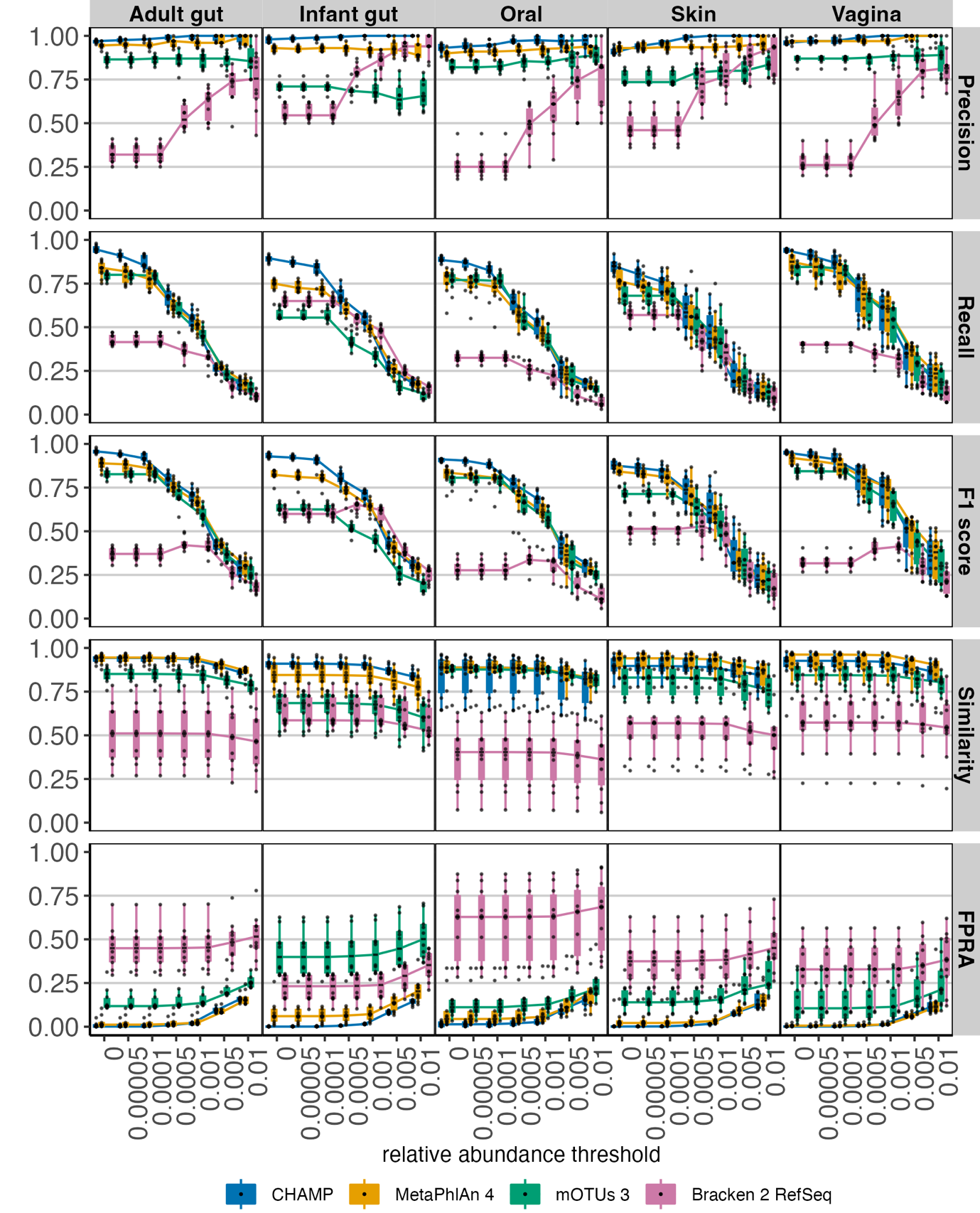


**Supplementary Figure 1.** Detection limits at species relative abundance cutoffs of 0.0005, 0.0001, 0.0005, 0.001, 0.005 and 0.001**.** 50 metagenomes were used and subset at the abundance cutoffs leading to 350 communities.

**
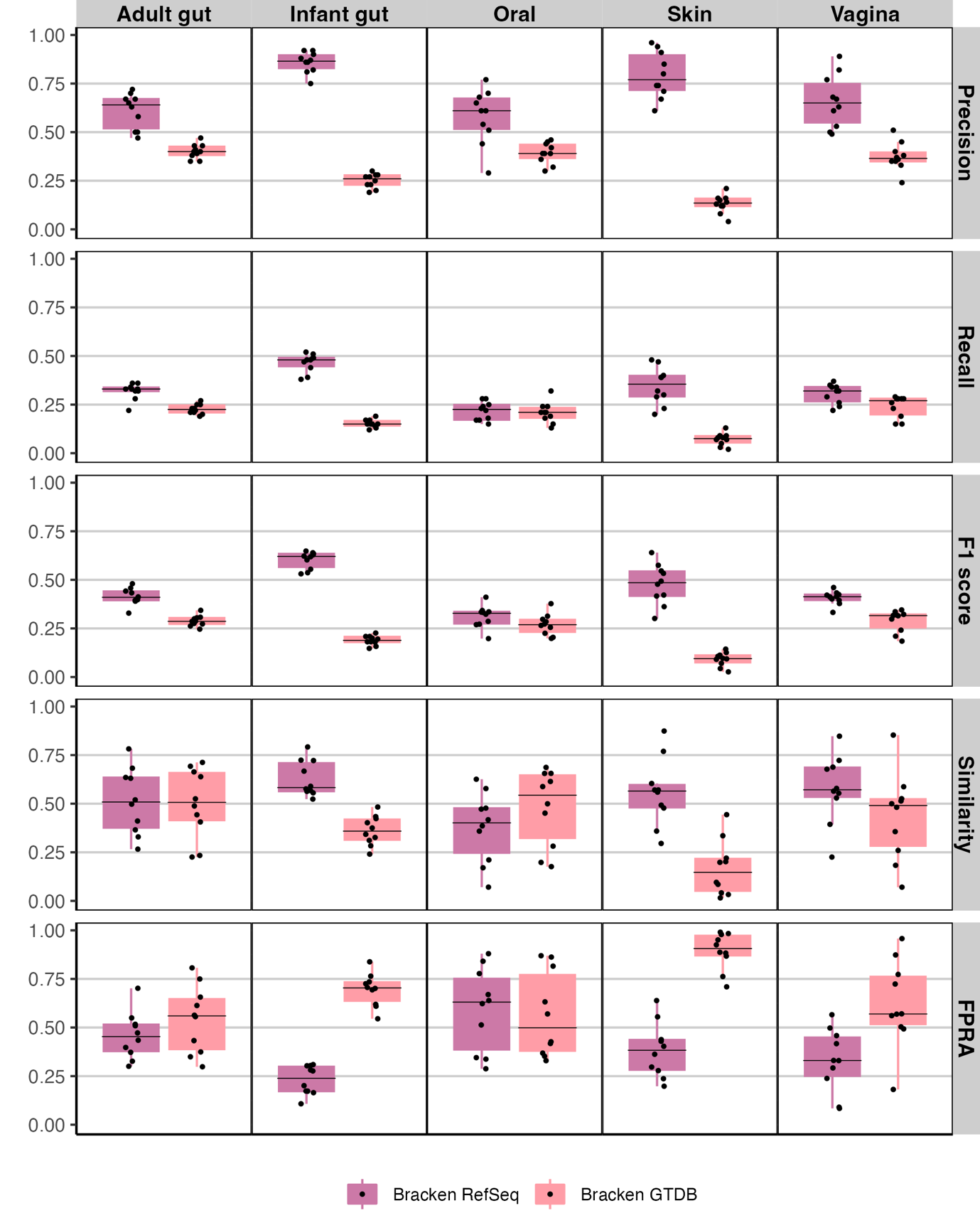
**

**Supplementary Figure 2.** Evaluating Bracken 2 with RefSeq vs GTDB r214 on key benchmarking metrics across 5 human body communities. 50 metagenomic communities (10 per body site) comprising 100 prokaryotic species each were used.

**
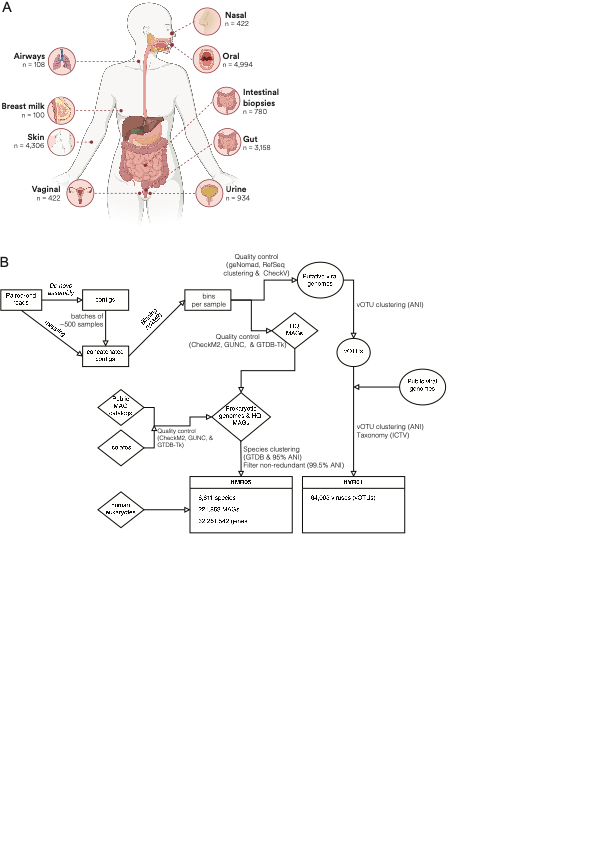
**

**Supplementary Figure 3. (A)** Overview of metagenomic samples per body sites used to generate new MAGs in this study. **(B)** Workflow to generate the Human Microbiome Reference (HMR05) and the Human Viral Reference (HVR01). Metagenomic samples were assembled *de-novo* and contigs were binned using VAMB in batches. HQ-MAGs and putative viral genomes were identified directly from bins. HQ-MAGs were combined with public catalogs, taxonomically annotated, and clustered to create the non-redundant MAG catalog. Putative viral genomes were clustered into vOTUs and combined with public viral genome repositories resulting in 64,003 vOTUs.

**
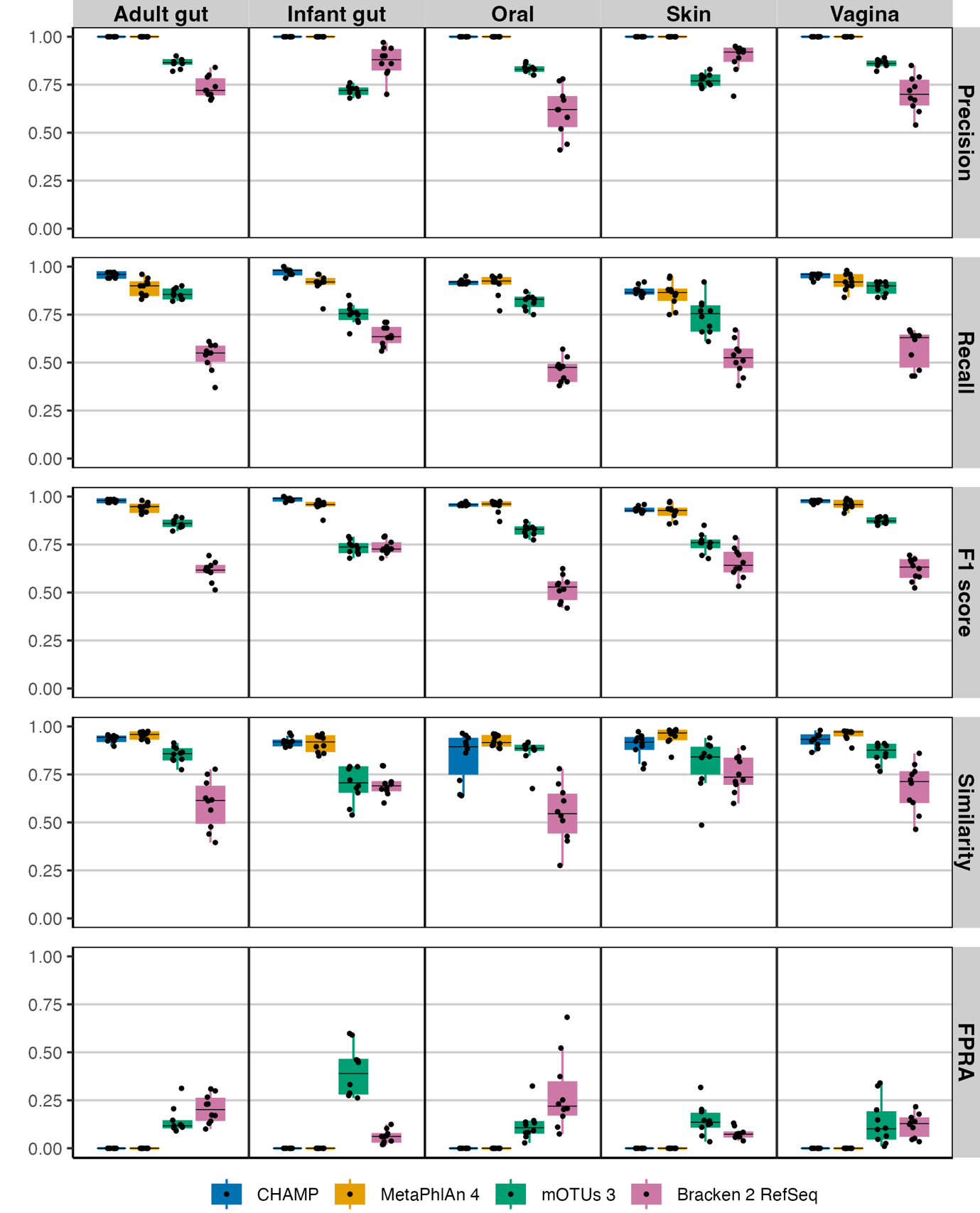
**

**Supplementary Figure 4.** Genus-level evaluation the taxonomic profilers CHAMP, MetaPhlAn 4, mOTUs 3, and Bracken 2 on key benchmarking metrics: precision, recall, F1 score, similarity, and FPRA across five human body communities. A total of 50 metagenomes (10 per body site) each with 100 prokaryotic species were simulated using CAMISIM.

**Supplementary Tables**

**Supplementary Table 1.** Genome accessions used for *in silico* metagenome simulations across body site communities

**Supplementary Table 2.** Genome accessions used for eukaryotes *in silico* metagenome simulations

**Supplementary Table 3.** Genome accessions used for virome *in silico* metagenome simulations

**Supplementary Table 4.** Overview of benchmarking metrics for *in silico* body site metagenome communities across profilers

**Supplementary Table 5.** Runtime and maximum memory comparisons (in Gb) comparisons across profilers for one *in silico* gut adult metagenome community (2.1 Gb). All profilers were run on default settings in a 1 compute node (40 processors per node) HPC.

**Supplementary Table 6.** Runtime and maximum memory comparisons (in Gb) comparisons between CHAMP and Phanta for bacteriophage profiling using one 50M *in silico* metagenome. All profilers were run on default settings in a 1 compute node (40 processors per node) HPC.
